# Seroprevalence and associated risk factors of Rift Valley fever in cattle and selected wildlife species at the livestock/wildlife interface areas of Gonarezhou National Park, Zimbabwe

**DOI:** 10.4102/ojvr.v87i1.1731

**Published:** 2020-04-08

**Authors:** Masimba Ndengu, Gift Matope, Musavengana Tivapasi, Davies M. Pfukenyi, Catherine Cetre-Sossah, Michel de Garine-Wichatitsky

**Affiliations:** 1 Department of Clinical Veterinary Studies, Faculty of Veterinary Science, University of Zimbabwe, Harare, Zimbabwe; 2 Department of Paraclinical Veterinary Studies, Faculty of Veterinary Science, University of Zimbabwe, Harare, Zimbabwe; 3 CIRAD, UMR ASTRE Animal Santé Territoires Risques Ecosystemes 2, Rue Maxime Rivière, Réunion, France; 4 UR AGIRs, Cirad, Campus International de Baillarguet, Montpellier, France

**Keywords:** Rift Valley fever, abortion, zoonosis, cattle, wildlife

## Abstract

A study was conducted to investigate the seroprevalence and associated risk factors of Rift Valley fever (RVF) infection in cattle and some selected wildlife species at selected interface areas at the periphery of the Great Limpopo Transfrontier Conservation Area in Zimbabwe. Three study sites were selected based on the type of livestock–wildlife interface: porous livestock–wildlife interface (unrestricted); non-porous livestock–wildlife interface (restricted by fencing) and livestock–wildlife non-interface (totally absent contact or control). Sera were collected from cattle aged ≥ 2 years representing both female and intact male. Sera were also collected from selected wild ungulates from Mabalauta (porous interface) and Chipinda Pools (non-interface) areas of the Gonarezhou National Park. Sera were tested for antibodies to Rift Valley fever virus (RVFV) using a competitive enzyme-linked immunosorbent assay (ELISA) test. AX2 test was used to assess differences between categories, and *p* < 0.05 was considered as significant. In cattle, the overall seroprevalence was 1.7% (17/1011) (95% confidence interval [CI]: 1.01–2.7). The porous interface recorded a seroprevalence of 2.3% (95% CI: 1.2–4.3), the non-porous interface recorded a prevalence of 1.8% (95% CI: 0.7–4.3) and the non-interface area recorded a seroprevalence of 0.4% (955 CI: 0.02–2.5), but the difference in seroprevalence according to site was not significant (*p* > 0.05). All impala and kudu samples tested negative. The overall seroprevalence in buffaloes was 11.7% (95% CI: 6.6–19.5), and there was no significant (*p* = 0.38) difference between the sites (Mabalauta, 4.4% [95% CI: 0.2–24] vs. Chipinda, 13.6% [95% CI: 7.6–23]). The overall seroprevalence in buffaloes (11.7%, 13/111) was significantly (*p* < 0.0001) higher than in cattle (1.7%, 17/1011). The results established the presence of RVFV in cattle and selected wildlife and that sylvatic infections may be present in buffalo populations. Further studies are required to investigate if the virus is circulating between cattle and wildlife.

## Introduction

Wildlife has often been perceived as a reservoir for some livestock diseases, and one major feature of human/wildlife interface areas is human encroachment into wildlife habitat. The drivers of this encroachment include climate change, deforestation, changing food patterns, the need for drinking water, the disposal of waste and the human population explosion. All of these together with changes in agricultural activities have profound effects on domestic livestock patterns that, in turn, influence wildlife behaviour and migratory patterns (Daszak, Cunningham & Hyatt 2001). One result is the increased opportunity for contact among humans, domestic livestock and wildlife (Deem, Karesh & Weisman 2001). This may result in increased transmission of zoonotic and anthropozoonotic diseases (Wolfe et al. 1998).The burgeoning increase in the consumption of bush meat in many parts of the world also provides opportunities for the spread of wildlife diseases to both domestic animals and humans (Karesh et al. 2005).

In Zimbabwe, one such disease that can infect livestock, wildlife and humans alike is Rift Valley fever (RVF), which is an arthropod-borne viral zoonosis with evidence of widespread occurrence in humans and animals in Africa and the Arabian Peninsula (Nanyingi et al. 2015). Rift Valley fever should be suspected when unusually heavy rains are followed by the occurrence of abortions together with fatal disease marked by necrosis and haemorrhages in the liver that particularly affect newborn lambs, kids and calves, concurrent with the occurrence of an influenza-like illness in farm workers and people handling raw meat (Daubney, Hudson & Granham 1931). According to the World Organization for Animal Health (OIE), RVF virus (RVFV) is an OIE high-impact transboundary pathogen with potential for bioterrorism and a setback to international livestock trade (OIE 2014). The RVFV is a phlebovirus belonging to the Bunyaviridae family of viruses (International Committee on Taxonomy of Viruses [ICTV] 2018), and has been isolated from over 30 species of mosquitoes in six genera (Anyamba et al. 2010; Linthicum et al. 2007). The first major outbreak of RVF in livestock in Zimbabwe was reported in 1978 (Swanepoel 1981), and since then enzootic and epizootic existence has been documented but unpublished (Department of Veterinary Services, Zimbabwe). Serological evidence of the infection in Zimbabwean wildlife has also been demonstrated (Anderson & Rowe 1998) and recently in cattle and wildlife by Caron et al. (2013). The role of wildlife in maintaining the disease during the interepidemic periods has been inconclusively speculated (Beechler et al. 2013; LaBeaud et al. 2011; Manore & Beechler 2015).

The aim of this study was to determine the seroprevalence of RVF in cattle and some selected wildlife species at three selected interface areas of the Gonarezhou National Park (GNP) in the South Eastern Lowveld (SEL) of Zimbabwe. The study further aimed to investigate risk factors, including exposure to wildlife, associated with the occurrence of the infection in cattle.

## Materials and methods

This study was conducted as part of a broader investigation into three zoonotic causes of abortion in cattle, namely, brucellosis, chlamydiosis and RVF. The results of two of the diseases, brucellosis and chlamydiosis, have been published (Gadaga et al. 2016; Ndengu et al. 2017a, 2017b, 2018).

### Study area

**FIGURE 1:**
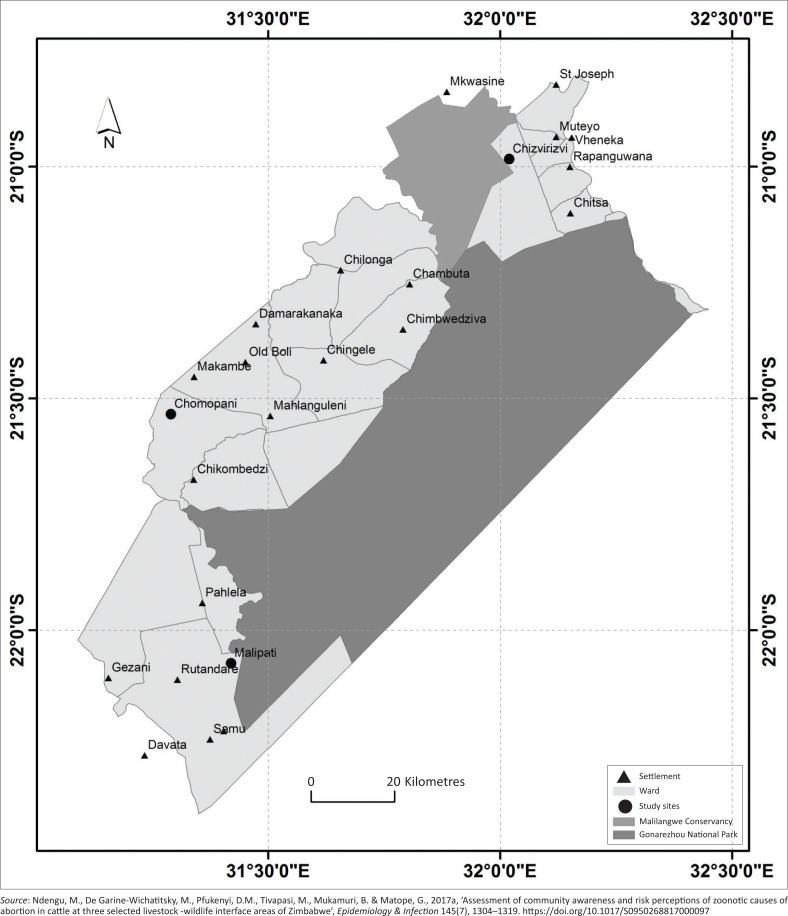
Map of the southeastern Lowveld of Zimbabwe showing the Gonarezhou National Park and the adjacent Malilangwe Conservancy. Note the three study sites represented by big black dots.

The study areas are located in the SEL of Zimbabwe in the agro-ecological Natural Region V, which is characterised by low elevations, high temperatures, and low and erratic rainfall (on average < 600 mm/year) (Gandiwa & Zisadza 2010). The selected study sites (Figure 1) comprised a livestock–wildlife interface, where there is contact between domestic and wild animals, and the non-interface areas, where interaction between domestic and wild animals is absent, as previously described (Ndengu et al. 2017a) and evidenced by the results of cattle and African buffalo global positioning system (GPS) tracking studies (Miguel et al. 2013; Zengeya et al. 2015). The livestock–wildlife interface included a porous interface with unrestricted livestock–wildlife contact and a non-porous interface with a fence preventing direct livestock–wildlife contacts (Ndengu et al. 2017a). The cattle populations in the three study sites are mainly crosses of the indigenous Mashona and Nguni breeds. These study sites are further described in detail below.

### Study sites and interface types

#### Porous livestock–wildlife interface

Malipati village (22°04’S, 31°25’E), located at the southern border of GNP on Sengwe communal lands (Gomo et al. 2012), was selected as the porous interface and the area has been described (Ndengu et al. 2017a). The village lies adjacent to GNP, and the veterinary fence, which was erected in 1975 to prohibit cattle/buffalo contacts (Dube et al. 2010), has been damaged extensively allowing free movement of livestock into the park and wildlife accessing human settlements. Cattle share grazing and watering sources with wildlife, particularly during the dry season when these resources are limited in the communal lands. It is therefore assumed that there is significant contact between humans, livestock including cattle and wildlife at this type of interface (Ndengu et al. 2017a).

#### Non-porous livestock–wildlife interface

Chizvirizvi village (20°59’S, 32°01’E), located on the periphery of the Malilangwe conservancy, was selected as the non-porous interface. The conservancy is located on the northern boundary of the GNP and is surrounded by a well-maintained game fence, which hinders direct contact between wildlife on one side of the fence and humans and livestock on the other. The conservancy is home to the full range of African wild ungulates occurring in the area, while on the other side of the fence, the Chizvirizvi village hosts livestock, mainly cattle, goats and sheep. The fence creates a physically defined linear interface, separating wildlife and cattle.

#### Livestock–wildlife non-interface

Chomupani communal lands (21°40’S, 31°19’E), located at least 15 km from the north western boundary of GNP, was the selected non-interface site. Wild ungulates are reportedly absent in the Chomupani area, and this site was considered to be a control site with no wildlife/livestock interactions as it was far away from the GNP.

### Study design

#### Cattle

A longitudinal study was designed targeting cattle populations in the three study sites of Chomupani, Chizvirizvi and Malipati, and populations of selected wild ungulate species in the neighbouring GNP. Communal cattle dip tanks were used as sampling frames in each of the selected study areas. In Zimbabwe, animal health regulations compel all cattle owners in rural areas to dip their cattle weekly during the rainy season and fortnightly during the dry season for control of ticks and tick-borne diseases (Chikerema, Matope & Pfukenyi 2013). For this reason, the government, through the Department of Livestock and Veterinary Services, has constructed communal dip tanks (plunge pools) in the rural animal health centres that are accessible by all farmers in the rural communities, with several villages sharing one dip tank. For each selected interface, one dip tank was chosen based on the history of compliance with previous research work and the appropriateness of its location to the interface type as defined in our study aims. Three dip tanks (Malipati, Chizvirizvi and Chomupani) were therefore selected representing the porous interface, non-porous interface and the non-interface, respectively. The cattle census was 1528 for Malipati, 1470 for Chizvirizvi and 2300 for Chomupani dip tanks. The minimum cattle sample size to be sampled was calculated using the formula:

$$n=[z^{2}\times p(1-q)]/e^{2},$$ [Eqn 1]

where *z* is the value from standard normal distribution corresponding to the desired confidence level (*z* = 1.96 for 95% confidence interval [CI]), *p* is the estimated prevalence and *e* is the desired precision (Dohoo, Martin & Stryhn 2003). We estimated an individual prevalence of 12% for RVF based on previous studies in the same area (Caron et al. 2013) and a 5% error margin at 95% confidence level. A minimum of 104 cattle per dip tank was therefore targeted per sampling session. Samples were recovered at each site in the wet and dry seasons that mainly occur in Zimbabwe from November to March and April to October, respectively.

#### Wildlife

Three wildlife species, the African buffalo (*Syncerus caffer*), greater kudu (*Tragelaphus strepsiceros*) and impala (*Aepyceros melampus*), were targeted for sampling south of the GNP in Mabalauta close to the porous Malipati interface. The species were selected on the basis of their reported mixing with livestock during grazing and at water points as confirmed by communities in previous studies (Ndengu et al. 2017a) and GPS radio-tracking studies for African buffaloes (Miguel et al. 2013; Zengeya et al. 2015). Another herd of African buffaloes at Chipinda, north of GNP, was also targeted. This herd lived far from target communities in a non-porous interface and was therefore assumed to be in minimal or no direct contact with domestic animals. Because of the cost of the capture process for wildlife, animals were conveniently sampled and the numbers sampled were limited. The numbers and procedures for wildlife immobilisation and sampling followed the description given in the permit granted by Zimbabwe National Parks and Wildlife Management Authority (permit N° 59(4) (a) & (b) 24/2015) and was performed by experienced licensed practitioners.

### Data collection

#### Cattle sampling

Each cattle dip tank was visited four times for sampling, as highlighted previously. Farmers enrolled their herd in the sampling protocol on a voluntary basis. Cattle were systematically randomly selected as they passed through the handling facilities of the dip tank. As cattle passed through the handling pen, each fourth mature (>1 year) and intact (if male) individual was targeted for sampling. The targeted individual would then be physically restrained and 15 mL – 20 mL of blood collected using coccygeal venipuncture and a 20 mL disposable plastic syringe and an 18-gauge needle. Upon collection, the blood was immediately transferred to 4 mL plain tubes. To allow clot separation, all blood samples were left to stand for approximately 15 min in the shade at ambient temperature (25 °C – 30 °C). Clotted blood samples were then centrifuged at 3000 *g* for15 min and 2 mL of serum were collected into cryo-tubes and stored in liquid nitrogen at −196 °C *en route* to the laboratory where they were stored at −20 °C until the time of analysis. As each individual animal was being sampled, epidemiological data pertaining to that individual was simultaneously collected. Among the data collected were the date and season of sample collection, interface type, owner of the animal and the village of origin, the sex and the parity in case of females and any history of previous abortion(s). All sampled animals were ear-tagged to avoid resampling them on subsequent visits.

#### Wildlife sampling

Within GNP, two buffalo groups were selected by aerial spotting from a helicopter in an area as close as possible to the porous park border (Mabalauta area), and in the northern part of the park (Chipinda area) closer to the non-porous border, respectively. The buffaloes were immobilised using a standard protocol similar to Burroughs et al. (2006): one to four individuals were anesthetised via a dart gun from a helicopter using a combination of etorphine hydrochloride and xylazine. The kudus were immobilised using similar standard procedures (Burroughs et al. 2006), although they were darted from the ground after being driven into a boma structure, using pole syringes. The impalas were captured less than 2 km from Malipati dip tank, right at the edge of the porous interface, using nets followed by physical restraint without anaesthesia (Kock & Morkel 2006). Following immobilisation, blood was collected using jugular venipuncture and processed for laboratory analysis as described for cattle above. After sample collection from buffaloes and kudus, anaesthesia was reversed by injection of diprenorphine hydrochloride. The animals were released at the site of capture and monitored from the air or the ground until they recovered fully. Epidemiological data collected for each wild animal sampled included the date of capture, the age as estimated by dentition and the location using GPS. All sampled animals were classified as adults, subadults or juveniles.

#### Serological assays for the detection of Rift Valley fever virus-specific antibodies

Specific anti-RVFV antibodies were tested in serum samples using the commercial ID Screen® RVFV competition multi-species enzyme-linked immunosorbent assay (ELISA) (IDvet, Grabels, France [www.id-vet.com]) with a level of sensitivity and specificity of 91% – 100% and 100% (95% CI: 99.58% – 100%), respectively, based on livestock samples (bovine, ovine, caprine) (Comtet et al. 2010; Kortekaas et al. 2013). Based on competition, this test is able to detect specific RVFV antibodies regardless of the species tested (Gür et al. 2017; Moiane et al. 2017). The detection of RVFV-specific anti-N antibodies by ELISA indicates exposure to RVFV either by natural infection or by vaccination. Briefly, wells on the ELISA plate were coated with a recombinant RVFV nucleoprotein. Samples to be tested and the controls were added to the microwells. Anti-nucleoprotein antibodies, if present, would form an antibody–antigen complex, which masks the nucleoprotein epitopes. An anti-nucleoprotein-peroxidase conjugate was added to the microwells to fix the remaining free nucleoprotein epitopes forming an antigen-conjugate-HRP complex. After washes aiming to eliminate excess antigen–conjugate complex, the substrate solution was added and the resultant colouration depended on the quantity of specific antibodies present in the test sample. The optical density (OD) was measured using an ELISA microplate reader at 450 nm. For each sample, the competition percentage (S/N%) was calculated using the formula: S/N% = OD sample/OD negative control × 100. An SN% of less than or equal to 40% was considered positive for RVFV antibodies.

### Statistical analysis

#### Descriptive statistics

Data recording and editing were performed in Microsoft Excel^®^, and the descriptive statistics were performed using EpiCalc 2000 version 2. The overall number of seropositive animals was calculated from the total number of samples tested over the study period and expressed as a percentage. For cattle, seropositivity was examined in relation to interface type, sex, parity, abortion history and season, while seropositive wild animals were examined in relation to species, age and site. Interface type, sex, parity, abortion history and season categories were generated as follows: three for interface type (porous, non-porous and non-interface), two for sex (females and males), four for parity (heifers, 1–2, 3–4 and ≥ 5), two for abortion history (history of abortion and no history for abortion) and two for season (wet and dry). For wildlife, species, age and site categories were as follows: three for species (buffalo, kudu and impala), three for age (juveniles, subadults and adults) and two for site (Mabalauta and Chipinda). Descriptive statistics on abortion history, parity and season were restricted to female cattle and because of a small sample size, seasonal and interface type descriptive statistics were not assessed for male cattle. The chi-square test was used to measure differences between categories, and values of *p* < 0.05 were considered as significant.

### Ethical considerations

All standard ethical consideration were followed with strict observation to the five degrees of animal welfare freedom adhered to.

## Results

### Cattle seroprevalence

A total of 1011 cattle were sampled with 46.7% from the porous (Malipati), 28.2% from the non-porous (Chizvirizvi) and 25.1% from the non-interface (Chomupani) areas.

The distribution of the sampled animals and their RVF seroprevalence according to interface, sex, abortion history and season is shown in Table 1. The overall seroprevalence was 1.7% (95% CI: 1.01–2.7) and it varied according to interface, but the differences were not significant (*p* > 0.05). All positive cattle were females, but again the difference according to sex was not significant (*p* = 0.27). Similarly, the difference in seroprevalence according to abortion history in cows was non-significant (*p* = 0.44). In contrast, the wet season recorded a significantly (*p* = 0.0002) higher seroprevalence compared with the dry season (data are for female cattle only).

**TABLE 1:** Distribution of Rift Valley fever seroprevalence in cattle according to different categories.

Category	Level	No. tested	Positive	Seroprevalence* (%)	95% CI
	All animals	1011	17	1.7	1.01–2.7
Interface	Porous (Malipati)	472	11	2.3^a^	1.2–4.3
	Non-porous (Chizvirizvi)	285	5	1.8^a^	0.7–4.3
	Non-interface (Chomupani)	254	1	0.4^a^	0.02–2.5
Sex	Female	897	17	1.9^a^	1.1–3.1
	Male	114	0	0.0^a^	0.1–4.1
Abortion history†	Yes	138	1	0.7^a^	0.04–4.6
	No	750	16	2.1^a^	1.3–3.5
Season‡	Wet	325	14	4.3^a^	2.5–7.3
	Dry	572	3	0.5^b^	0.1–1.7

CI, confidence interval.

* , Figures with a different superscript letters in the same category are significantly different at *p* < 0.05.

† , Abortion history was not given for nine cows.

‡ , Seasonal comparisons were done on female cattle only.

### Wildlife seroprevalence

A total of 161 samples were collected from wild animals: 111 buffaloes, 32 impalas and 18 kudus. Table 2 shows a summary of the sampled animals and their seroprevalence according to site. All impala and kudu samples tested negative. The overall seroprevalence in buffaloes was 11.7% (95% CI: 6.6–19.5) and there was no significant (*p* = 0.38) difference between the sites (4.4% Mabalauta vs. 13.6% Chipinda).

**TABLE 2:** Distribution of Rift Valley fever seroprevalence in wildlife according to site and species.

Site	Species	No. tested	Positive	Seroprevalence (%)	95% CI
Mabalauta	Impala	32	0	0.0	0.0
	Kudu	18	0	0.0	0.0
	Buffalo	23	1	4.4	0.2–24.0
	Overall	73	1	1.4	0.1–8.4
Chipinda	Buffalo	88	12	13.6	7.6–23.0
**Grand total**		**161**	**13**	**8.1**	**4.6–13.7**

CI, confidence interval.

The overall seroprevalence in buffaloes (11.7%, 13/111) was significantly (*p* < 0.0001) higher than in cattle (1.7%, 17/1011).

## Discussion

Rift Valley fever is an important arthropod-borne zoonosis that affects wildlife, livestock and humans with serious consequences. The present study aimed at determining the seroprevalence of this disease in cattle at three selected wildlife/livestock/human interface areas as well as its seroprevalence in selected wildlife species, namely, buffalo, impala and kudu. These selected wildlife species have been shown to spatially interact with some of the cattle in the study, particularly at the porous interface. Apart from the direct physical contact, which is aided by the disruption of fences on the porous interface, cattle and wildlife are vulnerable to the same mosquito vectors of the RVFV (Linthicum et al. 1984; Meegan et al. 1980) and therefore interspecies transmission of the disease can still occur in spite of the physical barriers that prevent direct contact.

Our study did not focus on the clinical diagnosis of RVF but rather on evidence of exposure to the virus as determined by the presence or absence of RVFV-specific antibodies (seroprevalence). The serological test used, the competitive ELISA, has been shown to have a sensitivity of 91% – 100% and a specificity of 100% (95% CI: 91.24% – 100%) in cattle, horses, sheep, goats, cats, dogs and humans (Comtet et al. 2010; Kortekaas et al. 2013). In the study areas, vaccination against RVF is not practised and, thus, the results obtained are likely to indicate natural exposure to RVFV infection. Given the specificity range above, false positivity could not be ruled out given the low number of positive animals, but the fact that the disease has been demonstrated in the same areas before (Caron et al. 2013), coupled with absence of vaccination, likely indicates true positivity.

Our results show an overall low prevalence of the disease in cattle with no statistical differences between the three interface types. Previous studies in Zimbabwe, although scant, have demonstrated serological evidence of RVFV in cattle and wildlife (Anderson & Rowe 1998; Caron et al. 2013, 2016). The last official outbreak of RVF in Zimbabwe was recorded in 2001 (OIE 2014), although Sinyangwe (2013) reported that Zimbabwe had another outbreak in 2011, which was not reported to the OIE. The low seroprevalence in this study is, however, in contrast to the findings of Caron et al. (2013), who reported an overall higher seroprevalence of 12.1% from samples collected in 2008 in the same study areas. The seroprevalence of RVF was also higher in the wet season, compared with the dry season. This is because the mosquito vectors proliferate following heavy rains that are characteristic of the wet season (Linthicum et al. 1999; Swanepoel & Coetzer 2006). Our results, which indicate that exposure to wildlife is not a risk factor for RVFV infection in cattle, are, however, in contrast to those of Caron et al. (2013), who reported a significantly higher seroprevalence (18.3%) in the porous interface compared with the non-porous (8.5%) and non-interface (7.7%) areas. The fact that the seroprevalence in buffaloes was significantly higher than that in cattle can be attributed to two factors. Firstly, as suggested elsewhere (Beechler et al. 2013; Manore & Beechler 2015), there is a very low rate of subclinical circulation of RVFV in buffalo in the Kruger National Park (KNP), and secondly, the intermixing of buffalo populations from the parks that constitute the Great Limpopo Transfrontier Conservation Area (GLTCA) presents an opportunity for infection to be carried across national boundaries, especially given the history of occurrence of the disease in the KNP of South Africa (Beechler et al. 2013; Pienaar & Thompson 2013). In our study, the buffalo herd in Chipinda, farthest from the cattle, had a much higher seroprevalence of RVFV antibodies than the herd in Mabalauta, closest to the domestic livestock. This tends to suggest that the cryptic transmission cycles of the virus occur within the buffalo populations.

In our study, both kudus and impalas were detected as being seronegative for RVFV. There are no previous reports of the disease in kudus in Zimbabwe and only one out of 801 impalas tested positive for RVFV in the study by Anderson and Rowe (1998). Rift Valley fever virus, however, has been demonstrated in kudus in neighbouring South Africa from the 2010 outbreaks (Pienaar & Thompson 2013), proving that the species is susceptible. The fact that there was no serologic evidence of infection in these species is quite surprising given the fact that they do share the same ecosystem with the positive buffalo populations. The absence of evidence of infection in the two species could be an indication of an interepidemic status where the species play no role in virus maintenance or it could be because of the small sample size or even predation of weaker animals because of infection.

In conclusion, our study provided evidence that RVF is present in the SEL of Zimbabwe in both cattle and buffalo populations, with cattle having a low level of infection and buffaloes having a moderate level. Proximity to wildlife is not a risk factor to cattle and vice versa as differences in seropositivity between study sites were not significant. The kudus and impalas were all negative for infection in spite of sharing the same ecosphere as the buffaloes. The level of infection in cattle is too low to warrant any preventative measures such as vaccination. However, surveillance of the disease by the Department of Veterinary Services and the Ministry of Health under the One Health concept should continue as the disease is an important zoonosis. Further studies, including prospective cohort studies, should focus on investigating the possible role of buffaloes in the epidemiology of the disease in cattle.
